# Evaluating strengths and opportunities for a co-created climate change curriculum: Medical student perspectives

**DOI:** 10.3389/fpubh.2022.1021125

**Published:** 2022-10-24

**Authors:** Irene Liu, Benjamin Rabin, Madhu Manivannan, Emaline Laney, Rebecca Philipsborn

**Affiliations:** ^1^Emory University School of Medicine, Atlanta, GA, United States; ^2^Department of Internal Medicine, Brigham and Women's Hospital, Boston, MA, United States; ^3^Department of Pediatrics, Emory University School of Medicine, Atlanta, GA, United States; ^4^Gangarosa Department of Environmental Health, Rollins School of Public Health, Atlanta, GA, United States

**Keywords:** climate and health education, co-creation, Planetary Health, climate health, medical students, curriculum evaluation

## Abstract

**Introduction:**

Medical trainees are front-line workers in our worsening climate and health crisis. A movement is underway to teach medical students essential climate change and health content. Few evaluations of climate and health curricula exist to support ongoing curricular development, innovation, and improvement. This study explores student perspectives on climate change and health content and delivery post-implementation of a climate change and health curriculum that was co-created by students and faculty and integrated across 16 months of pre-clinical coursework at Emory University School of Medicine.

**Methods:**

The authors conducted focus groups with the inaugural cohort of students to receive the climate and health education content at the conclusion of their preclinical curriculum. The focus groups elicited student perspectives across four domains: (i) prior perceptions of climate change and health, (ii) current attitudes about climate change and health, (iii) reflections on the existing curriculum, and (iv) opportunities for the curriculum. In this qualitative evaluation, the authors coded focus group transcripts using an inductive content analysis approach.

**Results:**

Out of 137 eligible students in the cohort, 13 (9.5%) participated in the focus groups. Implementation strategies that students valued included contextualization and integration of climate content within existing topics and student representation through the co-creation process. Students recommended bolstering small group sessions and case-based learning to build relevant history and physical examination skills as well as creating interprofessional and community-based opportunities.

**Discussion:**

This evaluation offers in-depth student perspectives of our climate and health curriculum. Opportunities exist to synergize climate and health education with broader transformations in medicine toward health promotion and sustainable, climate-ready healthcare. From the input of focus groups, the authors derive a framework for strengthening and extending curricular content.

## Introduction

Medical trainees are front-line workers in our worsening climate and health crisis. In response to the urgency of the climate crisis and the historical lack of climate change and health content in medical schools, students have motivated efforts in climate and health education (CHE) and spearheaded a movement to prepare themselves for the challenges ahead (1–3). Educating learners across the medical education spectrum about climate change and health is now recognized as an essential component of adapting the healthcare system and meeting healthcare needs in this era of climate change (4–6). Emerging CHE efforts range from isolated lectures to specialized electives and integrated curricula (7–10). Bolstered by calls from students, faculty, and professional organizations, many institutions are seeking to accelerate adoption of CHE or expand their existing CHE content and activities (11, 12).

Available literature proposes models for integrating CHE but without a consensus on best practices (13, 14). Few evaluations of implemented curricula exist and few resources elucidate learner perspectives on educational priorities and approaches to content delivery. Identifying acceptable and effective means to integrate CHE into medical curricula is a pressing need for students and faculty.

**Figure 1:**
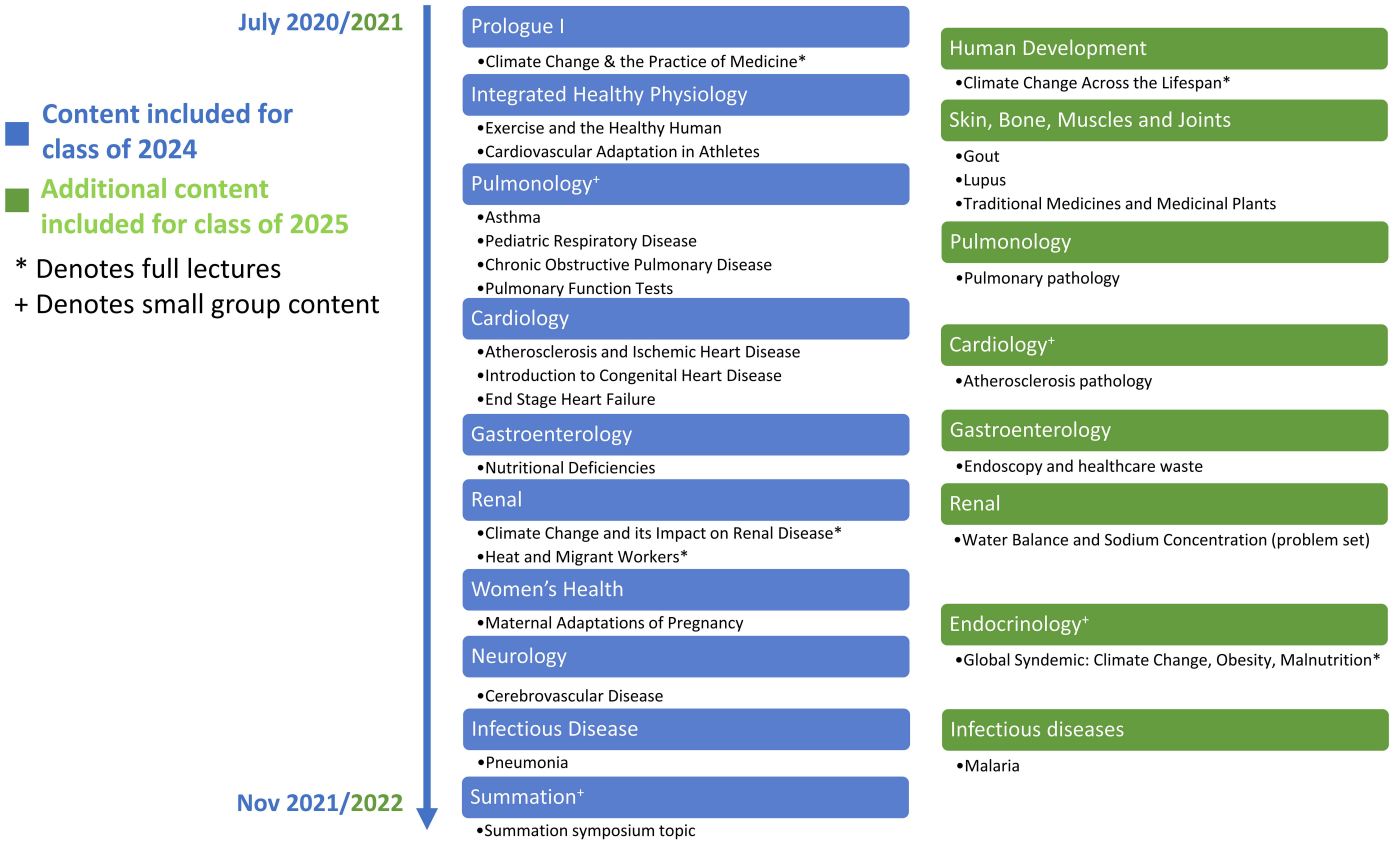
Climate change and environmental health content disseminated across pre-clinical courses and topics.

In the fall of 2020, our student and faculty team introduced a climate and health curriculum for all 139 students in the class of 2024 at Emory University School of Medicine (3). The curriculum integrates CHE across pre-clinical courses for first- and second-year students during the first 16 months, or pre-clinical coursework, of medical school (Figure 1). The role of students in envisioning and co-creating the curriculum as well as the initial learning objectives and the timeline of approval for the curriculum have been published previously (3).

Students, administrators, and lecturers were engaged to co-create and contextualize climate change and environmental health content within the foundational concepts of pre-clinical medical education. The curriculum includes both standalone lectures and integrated talking points and slides in pre-existing lectures as well as small groups discussions. In the first year of implementation, new CHE content was disseminated across lectures and small group sessions in more than 10 courses for the class of 2024. In some courses, student knowledge of CHE content was assessed through multiple choice questions at the discretion of individual course directors. This curriculum is ranked highly on the student-driven Planetary Health Report Card, which is a metric-driven evaluation tool launched in 2019 to evaluate CHE and sustainability initiatives at health professional schools (2).

At the conclusion of this pilot curriculum, we interviewed students in focus groups to better understand our first cohort's (i) prior perceptions of climate change and health, (ii) current attitudes about climate change and health for their careers, (iii) reflections on the existing curriculum, and (iv) identified opportunities for the curriculum. Participatory by nature, student focus groups extended our co-creation approach to curriculum evaluation. In this report, we present our analysis of student focus group transcripts and share perceptions and suggestions from the first cohort of students to receive our disseminated preclinical CHE curriculum.

## Methods

We randomly selected second year medical students (from the class of 2024) in the fall of 2021 to participate in the focus groups. Of 139 students in the cohort, 137 were eligible to participate. Two students were excluded because they are members of the CHE curriculum team (MM and IL). We aimed to include about 10% of eligible students, or 14 students, in two focus groups of 7 each. We randomly selected 14 eligible students and sent them emails informing them of the purpose of the evaluation and inviting participation. If invited students declined or did not respond within 1 week, we sent emails to additional randomly selected students until we reached our target enrollment of 14 students. Students were offered a $10 gift card and bamboo cutlery sets for participation. We obtained funding for these incentives from the Emory University Office of Sustainability Initiatives' General Sustainability and Social Justice Fund as a part of a grant to boost student participation in curriculum co-creation and to conduct a sustainable food and composting workshop. Emory University Institutional Review Board (IRB) did not require ethics approval for this curriculum evaluation.

We created an interview guide with probing questions related to our four domains of interest: (i) prior perceptions of climate change and health, (ii) current attitudes about climate change and health for their careers, (iii) reflections on the existing curriculum, and (iv) identified opportunities for the curriculum (Supplementary material). MM and IL each moderated one of the focus groups. As peers and members of the cohort to receive the curriculum, MM and IL were selected to reduce the potential power differential between moderators and participants. The moderators allowed participants to drive the conversation, using the questions only when needed to stimulate conversation and ensure exploration of each domain. Participation was voluntary and confidential. Responses were recorded anonymously. Participants gave verbal consent for audio-recording. The duration of the two focus groups ranged from 48 to 52 min.

We transcribed the focus group recordings using an online transcription service (scribie.com) (15). Two team members (IL and BR) independently verified the transcriptions, listening to the recordings and ensuring fidelity in the automated transcription. IL and BR independently analyzed and coded the transcripts using an inductive content analysis approach (16, 17). No *a priori* codes were used. Using an open coding process to identify phrases of meaning in the text, IL and BR assigned codes and grouped codes under the relevant domain. IL and BR met with RP to review and refine codebooks, organize codes into themes, and revise themes through an iterative process using in-depth discussions and comparisons of thematic relationships (16).

## Results

Between October 25th and November 1st, 2021, we conducted two focus groups with 6 and 7 participants, respectively. In total, 28 students were sent email invitations to participate in the focus groups. Of the 28 invited students, 14 agreed to participate, 11 had no response to two follow up emails in one week, one had a scheduling conflict, one opted not to participate because s/he did not attend lectures, and one did not want to participate with no reason stated. Of 14 students who agreed to participate, one was prevented from attending because of illness. The 13 who participated represent a sample of approximately 9.5% of the cohort to receive the inaugural disseminated CHE curriculum. Table 1 presents a summary of focus group discussions with themes, codes, and illustrative quotations (Table 1).

**Table 1:** Student focus group evaluation: Perceptions of climate change and a co-created and disseminated pre-clinical climate change and health curriculum across four domains.

**Themes**	**Codes**	**Quotations**
**Domain 1: Students' prior perceptions of climate change and health**		
Missing link between climate change & medicine	Knew the science	“Knew about the science of climate change…”; “I had a broad public health view.”
	Not patient health	“I don't think I knew as well about how it related directly to patient health.”
	Not an expected focus in the medical curriculum	“I expected to learn about social determinants of health…but I didn't expect climate change to be a part of that.”
	Important, but abstract	“I also thought of it very abstractly, almost in a different world to like, oh…worldly societal things that are important to me, and it was very separate.”
Variable level of engagement	Do one's part	“I did try to…do my part…reduce single-use products and not drive as much.”
	Reflecting prior exposure to sustainability	“As an undergrad…it was weaved into the curriculum or just the culture with peer health mentors that they had and a lot of sustainability efforts.“
	Not an advocate	“I wouldn't say I was super big into being an advocate.”
**Domain 2: Current attitudes about climate change and health in terms of their careers as doctors**		
Believe that climate change matters for patients and counseling	Affects history-taking	“We need to be thinking about those questions when we're talking to a patient.”
	Context for exposures	“You have to take into the context what their exposures are on a daily basis.”
	Useful in an upstream way	“Informing people why the situation is the way it is, is upstream to helping them decide to make change later on through their voting.”
	Location/context matters	“I think just with being in the South…seeing some of the air quality difference…the warmer climate, it seems to be almost dramatic.”
Working through application of climate and health knowledge in future clinical practice	Convergence of work and life	“Thinking about caring for patients while processing that personal experience [with climate disasters], it is all coming together.”
	Frustration at individual limits	“One of my challenges is…I don't know if my actions will make a difference.”
	Uncertain how to apply	“But one piece that's missing for me for me is like, 'What does it mean that I know this now?”'
Perceive need to engage through non-clinical activities	Advocacy	“A lot of the big changes that we need to do to fix the issue won't really happen unless we do things on a policy level.”
	Research	“There's not decades or there's not centuries of research…on climate change.”
**Domain 3: Reflections on the existing climate change and health curriculum**		
Enhanced awareness and applications in multiple domains	Medical waste	“The only one thing I can see after the case is… the post-surgery clean-up… [I] wouldn't have even looked at without…the lecture we have on how much medical waste is generated on a daily, annual basis.”
	Personal and professional growth	“…[Climate change] becomes something you consider in your daily practice, too, and hopefully moving forward as a physician.”
	Leadership	“It will be for the chance where we'll be in positions where we have opportunities to make an impact.”
	Patient health	“One of the biggest takeaways has been the application to patients.”
	Communication skills	“The most valuable part was how to talk about it.”
	Health equity	“When they showed the map with the red-lining, and how it overlapped perfectly with the map of the high incidence of heatstroke and the ambient temperature being higher, I think that really impacted me…”
Approach to content delivery matters	Meet the needs of different learners	“For 95% of the class, having key takeaway points is gonna do the trick …[but] one of the goals…is to inspire that 5% to do more…because we're gonna need that 5%, right?”
	Provide examples	“The more concrete an example or practical solution that they can give in those lectures is most meaningful.”
	Integrate seamlessly	“[When content] is weaved in and out through all the different lectures, it helps to kind of make it into more easily digestible bits. And also you can see more clearly how it impacts all these different areas of health.”
	Provide a frame/anchor	“It becomes a thing that helps you remember the other thing.”
	Faculty attitudes matter	“If the lecturer doesn't care, we can feel it.”
	Focus on facts rather than politics	“I really like how … it wasn't…there to sway people on climate change …instead…the focus of the lecture is more so like, this is climate change, this is why it's happening, this what we need to do.”
Perceived as more intuitive or deprioritized	Content is intuitive	“Part of it felt a little bit intuitive or kind of, ‘Oh, I probably know what they're gonna say,”'
	Traditional medical learning takes priority	“I care about this stuff, and…early on, when we were overwhelmed with anatomy, it was like, I am not even looking at this PowerPoint.”
**Domain 4: Student-identified opportunities for the curriculum**		
Opportunities in non-didactic spaces	History and physical	“Maybe redoing how we take physical exams incorporating more questions…and incorporating more environmental risk factors in the questions we ask.”
	Translate to rotations	“If I'm a primary care doctor, how this gets integrated into my decision-making.”
	Case reports	“Having that tied to a case would be just engaging and nice.”
	Community learning	“Incorporating some opportunities…[with] organizations that might be doing this work.”
	Small group	“I would really like to see climate change being more integrated into a small group. I'm also not a lecture watcher.”
	Include students	“If you invited students to give presentations on topics they cared about…people would be more engaged.”
	Career development	“Things to think about when you're looking at a residency, or an employer down the line, about if these are really important values to you.”
Role and modalities of assessments	Reflections	“Reflections…would allow people to kind of explore what they find interesting...”
	Student motivation	“Including test questions would probably not really motivate people.”
	Perceived yield	“It's like, that's not gonna be on step, that's not gonna be on the test.”

### Domain 1: Students' prior perceptions of climate change and health

Although students had engaged to different degrees with climate change prior to medical school matriculation (e.g., in their personal life or in their community), they were largely unaware of the links between climate change and medicine. They did not need or want convincing of the “science of climate change” and were well-versed in climate change basics. They had not expected, however, that climate change would be integrated into the medical curriculum. Summarizing a common stance, one student expressed lack of knowledge on “how it related directly to patient health.” Though some incorporated environmental sustainability in their personal lives (e.g., reducing single-use products) or came from undergraduate programs with a culture of sustainability, most students had not previously engaged in climate advocacy.

### Domain 2: Current attitudes about climate change and health in terms of their careers as doctors

At the conclusion of our curriculum, there was broad consensus on the relevance of climate change not only to public health but also to medicine. Some students identified specific ways that the content can be applied to patient care, especially to patient counseling and history-taking, with one noting, “We need to be thinking about those questions when we're talking to a patient.” Others still grappled with the application of climate change and health knowledge to clinical encounters, struggling with how they will translate the pathophysiologic concepts they had learned in the classroom to patient histories, clinical assessments, and care plans.

Students discussed the ways in which the curriculum influenced their own perceptions of their roles as health professionals. They perceived a need for physicians to engage in non-clinical realms, voicing the importance of more research and solutions at a policy level to address the climate crisis. One student expressed hopelessness, stating, “I don't know if my actions will make a difference.” Building upon the theme of individual constraints, upstream and policy changes were mentioned at several points as requisites to undergird the actions of individuals.

### Domain 3: Reflections on the existing climate change and health curriculum

Three themes emerged as students reflected on the preclinical climate and health curriculum. First, students agreed that the curriculum opened their eyes to climate change and health challenges (e.g., medical waste, patient health, and health equity) and avenues through which they could address these (e.g., by contributing to personal and professional growth as well as leadership and communication skills). Reflecting on medical waste, one student opined, “I've gone to two surgeries now where the only thing I can see after the case is over is all of the post-surgery clean-up.” The transcendence of climate change across personal and professional realms was noted: “[Climate change] becomes something that you consider in your daily [personal] practice, too, and hopefully moving forward as a physician.”

Second, students' preference was for content to be “weaved in and out through all the different lectures.” This integrated delivery method helped them to see “how it [climate change] impacts all these different areas of health.” While we aimed to contextualize our curriculum and anchor CHE within existing preclinical topics, for some students, CHE served as the more tangible scaffold for traditional medical concepts that otherwise seemed abstract or less immediately relevant: “A thing that helps you remember the other thing.” Students recalled the relationship of climate change with familiar social determinants of health, while appreciating climate-driven pathology, physiological changes to the body, and the myriad threats of climate-related exposures relevant across the organ system-based courses.

Students especially liked “concrete examples” linked to solutions and conversations about risk factors. As an example, “[Learning] about heat stroke and migrant workers…seemed like a very tangible thing…and also it taught us some direct things that we can encourage our patients…to take as precautions.” Students deemed faculty enthusiasm was crucial: “If the lecturer doesn't care, we can feel it.” They reflected that the curriculum should meet the needs of different learners, most of whom will want to know the foundational concepts, but some of whom will want to engage more deeply in research or advocacy or envision the subject as the future focus of their career. A student stated that although key takeaway points will suffice for many, “one of the goals…is to inspire that 5% to do more…because we're gonna need that 5%, right?”

Third, when students compared the CHE curriculum with core, traditional preclinical topics, many noted that anatomy and physiology take priority. This prioritization was not because the CHE curriculum was considered unimportant but due to time pressures. Students felt “overwhelmed with anatomy” and a “need to focus… on learning the physiology” to succeed. Others were less likely to pay attention to or study the integrated slides with CHE because they felt that the content might be more intuitive, reflecting a sentiment, “Oh, I probably know what they are going to say.”

### Domain 4: Student-identified opportunities for the curriculum

The majority of the preclinical CHE curriculum was lecture-based, and students proposed alternatives for more effective CHE content integration. Their ideas included incorporating content within the history and physical examination skills curriculum, 3rd and 4th year clinical clerkships, community learning opportunities, case-based learning, additional small group sessions, and research and advocacy opportunities. Students also requested space for career guidance: “Things to think about when you're looking at a residency or an employer down the line,” a manifestation of the importance of this topic for many. Students embraced the co-creation model and suggested further inclusion of peers in delivering content.

When asked directly about assessment modalities and whether their motivation to learn the content depended upon its incorporation in standardized medical licensing exams, students were not in agreement. Some felt that the topic lends itself well to reflection pieces or testing modalities other than multiple-choice questions. Some expressed the opinion that CHE is important (because of the urgency of the climate crisis) independent of its representation on tests. One noted that “including test questions probably would not really motivate people.” Another took a different stance, stating that content “that's not gonna be on the test,” will not get studied.

## Discussion

While students in these focus groups matriculated to medical school without awareness of the importance of climate change to medicine, our disseminated and co-created pre-clinical curriculum addressed this gap. Students explicitly valued strategies for CHE implementation: co-creating the curriculum, contextualizing CHE within existing topics emphasized in medical school, and integrating content throughout the curriculum.

Our curriculum leveraged these approaches due to practical considerations–saving time in a tight curriculum and updating the evidence base across organ systems. The student perspective offers added justification for building cohesion between CHE and pathophysiology, pharmacology, and traditional medical school topics: The contextualization becomes bidirectional. The CHE curriculum lends real-world meaning to the intensive and often unfamiliar concepts students learn in pre-clinical years.

**Figure 2:**
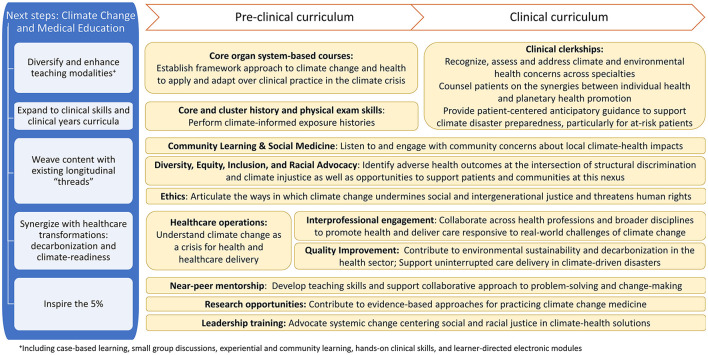
Climate change and environmental health in medical education: Curricular opportunities and learner outcomes informed by student perspectives. ^+^including case-based learning, small group discussions, experiential and community learning, hands-on clinical skills, and learner-directed electronic modules.

Integrating CHE into the curriculum also serves the needs of exceedingly practical students seeking to fulfill well-established and rigorous criteria to advance to the next stage on their journey of becoming a doctor. Not unique to CHE, the challenges of teaching students in lecture settings and incorporating structural determinants of health in the curriculum are well-documented (18, 19). Students overwhelmed by the quantity of information in lectures prefer a shift to clinical skills applications. This preference offers an important opportunity for CHE: Recognizing, assessing, and addressing climate-health impacts are vital skills for safeguarding patients and adapting health care in the climate crisis. Building on student input in these focus groups, Figure 2 summarizes next steps for our curriculum.

Many of our students acknowledged the burden of climate change in their future professional and personal lives, with comments indicating some level of climate grief and anxiety. Many also felt powerless to address environmental injustice and structural roots of health disparity. Giving students space for discussion and reflection, avenues for experiential learning and community engagement, tangible solutions that they can apply in clinical encounters, and strategies for effective advocacy may be important to support student mental health and well-being. That students valued the opportunity to provide input and requested even more engagement suggests that co-creation of CHE may enhance student buy-in.

Although students were randomized, our focus groups included relatively few participants. Despite much overlap in themes, the focus groups may not have reached thematic saturation and their views may not fully represent our cohort. The timing of these retrospective focus groups also may have resulted in recall bias. On the brink of dedicated study for the United States Medical Licensing Examination (USMLE) Step 1, students participated at a time of increased anxiety about test preparation and relatively little exposure to patient care. Finally, the perspectives offered by students in this study are ultimately responses to a specific CHE curriculum at one institution as part of a curriculum evaluation that may not be generalizable elsewhere.

Nevertheless, this evaluation offers in-depth student perspectives post-implementation of our CHE curriculum on *what* content students value and *how* they prefer to receive this content. These insights may benefit others seeking to create, implement and evaluate their own CHE curricula. The co-creation model is particularly suited to the important and urgent topic of climate change and health. The synergies of CHE with secure and sustainable care delivery as well levers of disease prevention—in this case climate and environmental exposures—offer many applications in the clinical years of medical school. Challenges remain, but the potential of CHE movements to influence transformation in medical education and healthcare delivery is real, pressing, and still largely untapped.

## Data availability statement

The original contributions presented in the study are included in the article/Supplementary material, further inquiries can be directed to the corresponding author/s.

## Ethics statement

Ethical review and approval was not required for this study in accordance with the local legislation and institutional requirements. Written informed consent from the program evaluation focus group participants was not required to participate in this study in accordance with the national legislation and the institutional requirements.

## Author contributions

All authors contributed to the design of the focus groups and this curricular evaluation. EL, BR, and RP co-created this curriculum. MM and IL created the focus group evaluation interview guide with mentorship from BR, EL, and RP. MM and IL conducted the focus groups. BR and IL analyzed the focus groups with RP. BR, IL, and RP drafted the manuscript. All authors reviewed and revised the manuscript. All authors agree to be accountable for the content of the work.

## Funding

Student incentives and bamboo server ware were obtained with a small grant from the Emory University Office of Sustainability Initiatives' General Sustainability and Social Justice Fund.

## Conflict of interest

The authors declare that the research was conducted in the absence of any commercial or financial relationships that could be construed as a potential conflict of interest. The handling editor declared a shared affiliation with one of the authors EL at the time of review.

## Publisher's note

All claims expressed in this article are solely those of the authors and do not necessarily represent those of their affiliated organizations, or those of the publisher, the editors and the reviewers. Any product that may be evaluated in this article, or claim that may be made by its manufacturer, is not guaranteed or endorsed by the publisher.
