# Supplementary material for: Evaluating strengths and opportunities for a co-created climate change curriculum: Medical student perspectives
Source: Front Public Health. 2022 Oct 24;10:1021125. doi: 10.3389/fpubh.2022.1021125 (PMC9638156; doi:10.3389/fpubh.2022.1021125)
Supplement: Supplementary file 1 [file Data_Sheet_1.PDF]

Appendix 1: Domains of Interest and Focus Group Questions for Climate and Health Education Curriculum Evaluation

Moderators used these questions as a guide to stimulate conversation only when needed. Participants were allowed to drive the conversation in focus group discussions.

Students' prior perceptions of climate change and health

- Did you know anything about climate change before you came to medical school?
- How important was climate change to you before you came to medical school?
- Prior to medical school, did you ever think about climate change from a human health lens?
- Did you expect to learn about climate change in medical school?

Current student attitudes about climate change and health for their careers

- How do you think climate change will impact your patients, if at all? Your future clinical practice, if at all?
- What has influenced your thoughts and attitudes about climate change?
- Do you think it is important for medical students to learn about climate change and its relationship to health?

Input on the existing climate change and health curriculum and relevance of topics covered

- Do you remember learning about climate change and health in the modules/courses over the last year? What was the most memorable? What was the most/least valuable?
- Has the climate change and health curriculum improved your understanding of the climate crisis as it affects health and healthcare delivery? (How?)
- Do you think learning about climate change and environmental health has affected your overall understanding of human health and disease?
- What other topics (around climate change and planetary health), if any, would you like to see incorporated into the curriculum?

Student-identified opportunities for the curriculum

- How/Would having climate change questions on United States Medical Licensing Exam (USMLE) Step 1 change your opinion of the importance of this topic?
- What is the most effective way of structuring the climate change curriculum content?
- Did you perceive any challenges when studying or learning climate content within the foundation's curriculum?
- Would you like to continue to see climate content incorporated into the clinical years of medical school?
